# USING FRAILTY SCREENING TO INFORM PREOPERATIVE MANAGEMENT OF HIGH-RISK SURGICAL PATIENTS

**DOI:** 10.1093/geroni/igae098.1560

**Published:** 2024-12-31

**Authors:** Fred Ko, Katherine Ritchey

**Affiliations:** Chair; Co-Chair

## Abstract

Frailty is a clinical syndrome of age-related decline in physiologic reserve and increased vulnerability to stressors. Frail older adults undergoing surgery are at higher risks for postoperative complications and mortality and increased health care costs. Thus, frailty has emerged as a robust predictor of poor surgical outcomes in vulnerable older patients. There have been immense interest and research for bench-to-bedside translation and integration of preoperative frailty screening into clinical practice. These efforts have centered on the development of preoperative frailty screening as a surgical risk-stratification tool and its application to guide preoperative management aimed to mitigate surgical risk in older adults. The Veterans Health Administration (VHA) has been recognized for its Surgical Pause initiative in which: (1) routine frailty screening is ascertained in surgical patients using the Risk Analysis Index (RAI) in order to identify those at the highest risk of postoperative complications, loss of independence, and mortality; and (2) multidisciplinary care strategies are implemented to reduce frailty-associated risks to enable reduction of potential complications before surgery in those identified as frail. In this symposium, we will provide a state-of-the-art look at the VHA experiences in: (1) validation of RAI as a preoperative risk-stratification tool in older adults; (2) implementation and integration of RAI screening in clinical practice; and (3) evaluation of RAI screening and multidisciplinary optimization board prior to surgery.

